# The emerging role of sarcopenia as a prognostic indicator in patients undergoing abdominal wall hernia repairs: a systematic review of the literature

**DOI:** 10.1007/s10029-020-02179-6

**Published:** 2020-04-16

**Authors:** S. T. Clark, G. Malietzis, T. N. Grove, J. T. Jenkins, A. C. J. Windsor, C. Kontovounisios, O. J. Warren

**Affiliations:** 1 Faculty of Medical and Health Sciences, University of Auckland, Grafton, Auckland, New Zealand; 2 Colorectal Surgical Unit, Chelsea and Westminster Hospital, Chelsea, London, UK; 3 Department of Surgery and Cancer, Imperial College, Chelsea and Westminster and the Royal Marsden Campus, Paddington, London, UK; 4 Department of Surgery, St. Mark’s Hospital, Watford Road, Harrow, Middlesex UK; 5 Department of Surgery, Royal Marsden Hospital, London, UK; 6 HCA Healthcare, London, UK

**Keywords:** Systematic review, Ventral hernia, Abdominal wall reconstruction, Sarcopenia, Body composition, Outcomes, Visceral obesity

## Abstract

**Background:**

There is strong evidence suggesting that excessive fat distribution, for example, in the bowel mesentery or a reduction in lean body mass (sarcopenia) can influence short-, mid-, and long-term outcomes from patients undergoing various types of surgery. Body composition (BC) analysis aims to measure and quantify this into a parameter that can be used to assess patients being treated for abdominal wall hernia (AWH). This study aims to review the evidence linking quantification of BC with short- and long-term abdominal wall hernia repair outcomes.

**Methods:**

A systematic review was performed according to the PRISMA guidelines. The literature search was performed on all studies that included BC analysis in patients undergoing treatment for AWH using Medline, Google Scholar and Cochrane databases by two independent reviewers. Outcomes of interest included short-term recovery, recurrence outcomes, and long-term data.

**Results:**

201 studies were identified, of which 4 met the inclusion criteria. None of the studies were randomized controlled trials and all were cohort studies. There was considerable variability in the landmark axial levels and skeletal muscle(s) chosen for analysis, alongside the methods of measuring the cross-sectional area and the parameters used to define sarcopenia. Only two studies identified an increased risk of postoperative complications associated with the presence of sarcopenia. This included an increased risk of hernia recurrence, postoperative ileus and prolonged hospitalisation.

**Conclusion:**

There is some evidence to suggest that BC techniques could be used to help predict surgical outcomes and allow early optimisation in AWH patients. However, the lack of consistency in chosen methodology, combined with the outdated definitions of sarcopenia, makes drawing any conclusions difficult. Whether body composition modification can be used to improve outcomes remains to be determined.

## Introduction

Sarcopenia is of increasing clinical interest in a number of complex surgical specialties, including surgical oncology, transplant surgery, trauma, emergency, and vascular surgery [1–10]. Sarcopenia is defined as a loss of skeletal muscle mass, with an associated reduction in muscle strength and functional capacity [11–13]. Recent studies have identified sarcopenia as an independent predictor of poor postoperative outcomes following major abdominal surgery, particularly in patients undergoing oncological resection. In such general surgical populations, sarcopenia has been associated with increased rates of infection, length of hospital stay, morbidity, mortality and readmission rates [3, 5–7, 14]. It has also been associated with an increased cost for all major surgeries, particularly in the immediate postoperative period [2, 15].

Initially, sarcopenia was defined as an age-related decline in skeletal muscle mass. However, studies now emphasise the importance of “biological age” over “chronological age” when considering the cause of muscle loss, as there are pathological states besides normal physiological ageing which are capable of inducing catabolism and muscle wasting, resulting in sarcopenia. Examples of this include liver cirrhosis, malignancy, chronic diseases, nutritional deficiencies and immunosuppression [2, 16, 18]. Despite the consensus that computerised tomography (CT) is a reliable means of measuring core skeletal muscle volume, considerable heterogeneity still remains regarding the diagnostic criteria for sarcopenia [2].

Ventral hernia repairs (VHR), of both primary ventral and incisional hernias, are one of the most commonly performed general surgical procedures worldwide, with approximately 350,000 cases performed annually in the United States of America and approximately 100,000 cases in the National Health System (NHS) England [19–21]. Complex abdominal wall reconstruction (AWR), whereby fascial closure and hernia repair are complicated by large hernia size, need for component separation, need for adhesiolysis or flap reconstruction, is a growing specialty in its own right, with 11–23% of all midline laparotomies being complicated by abdominal wall incisional hernias [22, 23]. Unlike many surgical specialties, AWR consists of primarily elective cases, allowing time for thorough pre-operative planning, multidisciplinary discussion and pre-optimisation, all of which are fundamental to optimising patient care and surgical outcomes. Pre-operative factors such as obesity, smoking, diabetes mellitus, previous tissue plane disruption, previous chemotherapy and liver disease have all been shown to negatively impact postoperative outcomes in patients undergoing major abdominal surgery, including VHR and AWR [20, 24, 25].

Unlike the known surgical risk factors mentioned above, the role of sarcopenia as a surgical risk factor is not entirely clear, with even less known about its specific role in VHR and AWR surgery [20]. The aims of this review are to synthesise the available literature on sarcopenia and to determine its impact on the postoperative outcomes of patients undergoing abdominal wall reconstruction surgery.

## Method

### Search strategy

A comprehensive systematic review of the literature was conducted by the first author (S.C.) under the guidance of a qualified medical librarian, in keeping with the Preferred Reporting Items for Systematic Review and Meta-Analysis (PRISMA) guidelines [26]. The electronic databases searched in this systematic review included Medline, Google Scholar and Cochrane Library, using the following search terms: (“sarcopenia” OR “core muscle” OR “body composition” OR “myopenia”) AND (“abdominal wall reconstruction” OR “ventral hernia repair” OR “hernia” OR “complex abdominal wall”) AND (“computerized tomography” OR “tomography” OR “CT-scan”) AND (“outcomes” OR “length of stay” OR “discharge” OR “readmission” OR “return to theatre” OR “complications” OR “morbidity” OR “mortality” OR “hernia recurrence” OR “SSO” OR “surgical site occurrence” OR “SSI” OR “surgical site infection” OR “infection”).

### Selection strategy

Articles were included if they were published between 1st January 2000 and 1st December 2019. Articles were initially included based on their title and abstract. All duplicates were then reviewed and studies that failed to adhere to the inclusion criteria were excluded. Subsequently, complete copies of the full-text were obtained and analysed before confirmation of inclusion. Studies that failed to meet the inclusion criteria were excluded, with a recorded reason. Finally, the search results were supplemented by a manual search of relevant reviews, alongside their references, to ensure that all eligible studies were included in this review.

Studies were eligible for inclusion if English was the full-text language. Patients had to be > 18 years old, preoperative CT scans had to be within 1 year of patients undergoing ventral hernia repair or abdominal wall reconstruction, and preoperative comorbidities and postoperative outcomes (including hernia recurrence) of patients had to be reported. Our primary outcomes of interest were the degree of lean muscle and ventral hernia recurrence. Secondary outcomes included length of stay (LOS), surgical site infection (SSI), surgical site reoccurrence, readmission, return to theatre and other post-operative complications. Assessment of lean muscle was limited to studies reporting radiological assessment methods, including CT, magnetic resonance and dual-energy X-ray absorptiometry. Studies were excluded if they were case reports, review articles or animal studies. Furthermore, only original and published studies were eligible for inclusion.

### Data extraction and analysis

Study inclusion was initially decided by SC and also discussed with TG and OW. If two papers reported on the same patient group, the larger, most recent, and highest quality publication was selected for inclusion. Discrepancies in data extraction were resolved by a third independent reviewer (GM). Selected studies were compared using a data table (Tables 1, 2, 3, 4) which included details on the number of patients in each study, study design, type of abdominal surgery, hernia characteristics, patient comorbidities, the outcome measures, follow-up period and the results of the surgery. The results were described in narrative analyses. Mendeley reference management software was used to manage citations (Mendeley Desktop v 1.19.4, London, UK).

## Results

### Search outcome

**Fig. 1:**
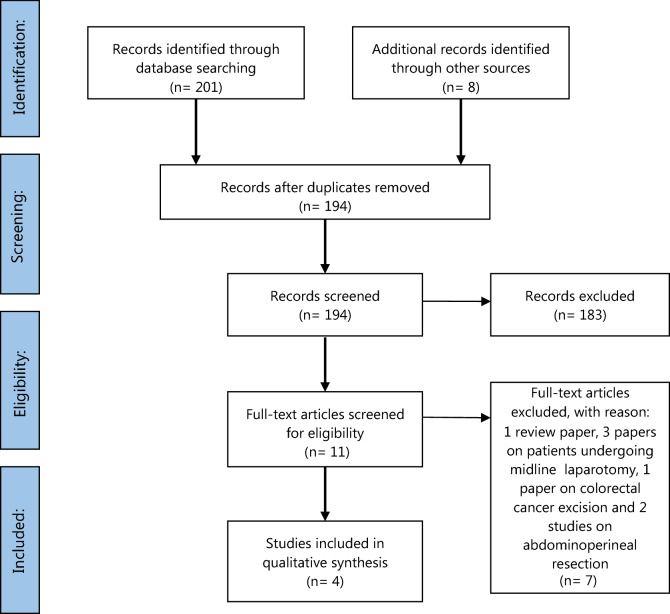
Flow diagram of literature review method

Overall, four studies were considered eligible for this systematic review, after applying our inclusion and exclusion criteria [20, 27–29]. The PRISMA flow diagram of the literature search process is shown in Fig. 1 and includes reasons for removal of studies.

### Participant population

Altogether, 1453 patients were enrolled in the four studies. 42.8% were males and the mean age of the patient population was 57.6 years. The mean BMI was 33.4 kg/m^2^. In total, 224 patients (25.4%) out of the participant population had sarcopenia. Participant characteristics are summarised in Table 1.

### Study characteristics

Three of the studies included in this systematic review were retrospective cohort studies [20, 27, 28]. Schlosser et al. authored the only prospective study in this review [29]. All four studies exclusively included patients undergoing ventral hernia repair and/or abdominal wall reconstruction [20, 27–29].

### Definition of sarcopenia

Image analysis of cross-sectional CT scans to measure core muscle area, and the diagnostic criteria used to determine sarcopenia differed between studies. The majority of studies used L3 as their chosen level for cross-sectional measurement on CT [20, 28, 29], whilst only one study used L4 as their chosen landmark [27]. Furthermore, two studies identified total abdominal muscle area (TAMA) by applying the skeletal muscle-specific Hounsfield Unit (HU) thresholds [20, 28]. The remaining studies manually outlined the psoas muscle on a semi-automated software to determine TPA [27, 29].

All four studies accounted for bone, vasculature and fat infiltration into muscle, by applying skeletal muscle-specific HU [20, 27–29]. Only one study did not normalise core muscle area by patient height [27]. All four studies stratified their study population into sarcopenic and non-sarcopenic patients [20, 27–29]. This stratification occurred either by quartile, in reference to study-specific cut-off values for the core-muscle area [27], or by gender-specific cut-off values for sarcopenia [20, 28, 29].

Only one study identified complications using the Clavien–Dindo classification [29], whilst the remaining three studies used various predefined complications [20, 27, 28]. The study characteristics of each eligible paper are outlined in Table 2.

### Hernia characteristics

There was considerable heterogeneity in recorded hernia characteristics between studies. Two studies measured mean hernia volume and average abdominal defect area [28, 29], whilst Barnes et al. only recorded median hernia volume [27]. Furthermore, only Siegal et al. utilised the modified Ventral Hernia Working Group (mVHWG) classification to grade hernia severity [20]. Three of the studies recorded each patient’s history of prior hernia repair [20, 28, 29], whilst only Barnes et al. and Seigal et al. specify if any concomitant procedures were performed [27, 28]. Hernia characteristics are summarised in Table 3.

### Postoperative outcomes

Study outcomes related to sarcopenia are summarised in Table 4. All four studies assessed postoperative complications [20, 27–29]. Overall, only two of these studies identified significant findings in terms of an increased overall risk of postoperative complications, particularly hernia recurrence and renal failure [27] and prolonged hospitalisation in sarcopenic patients [28]. Furthermore, after multivariate linear regression analysis, Barnes et al. identified a significant association between a reduction in lean muscle mass and post-operative complications (*p* = 0.04), with sarcopenic patients having a 5.3-fold increased risk of post-operative complications, e.g. hernia recurrence, relative to patients without sarcopenia [27].

All four studies assessed the length of stay following surgery, readmission to theatre, surgical site infections and surgical site occurrence [20, 27–29]. Schlosser et al. showed hernia recurrence to be associated with both previous hernia repair and contamination, despite no association with the presence of sarcopenia [29]. Interestingly, Siegal et al. identified no significant associations between the presence of sarcopenia and any post-operative outcomes. However, the study did identify a significant increase in patient odds of in-hospital morbidity (1.44), per 10 cm^2^/m^2^ reduction in muscle index, after adjusting for diabetes mellitus, critical care status and BMI [20].

### Mortality outcomes

Unlike the majority of studies in the literature assessing the impact of sarcopenia on surgical outcomes, mortality was not assessed in any of the studies in this review. This reflects the benign nature of hernia repair, relative to patients with malignancies or organ failure, undergoing surgery.

## Discussion

Recent studies have shown sarcopenia to be notably prevalent in adult study populations. In a recent systematic review, Shafiee et al. identified a 10% prevalence of sarcopenia in adults aged > 65 years old in the general population [30]. These values rise to 20–70% in cancer patients (dependent on tumour type) [30] and to approximately 27% in VHR and AWR patient populations [12, 31, 32]. To our knowledge, this is the first study to systematically review the role of sarcopenia on postoperative outcomes following VHR or AWR. We identified a mean prevalence of 25.4% in patients undergoing VHR. However, as highlighted by two of the studies included in this review [20, 28], this may be an underestimation of the true prevalence of sarcopenia in the general and in-patient population.

The majority of studies in the literature define sarcopenia using index cut-off values derived from studies of patients with cancer cachexia or liver cirrhosis [20, 28]. Malignancy and other severe diseases induce systemic inflammation, catabolite stimulation of skeletal muscle and promote reduced protein intake. This results in muscular atrophy, deconditioning and ultimately sarcopenia [14, 28, 33]. Sarcopenia is also seen to develop at a faster rate, with a more significant impact on health outcomes in malignant patients, relative to patients with benign conditions [14, 34]. This differs from “physiological” sarcopenia which is considered to be a part of the “frailty” syndrome, and therefore, does not require the presence of an underlying disease [14, 35]. Rather, it is most commonly attributed to increased age and demonstrates milder inflammatory levels, metabolic disturbances and functional impairment [14, 28]. Such differences in disease aetiology and subsequent indications for surgery suggest that these historical cut-off values may be inappropriate when assessing sarcopenia in a benign cohort of patients, such as those awaiting VHR or AWR.

In support of this idea, Seigal et al. performed a de novo analysis of muscle index as a continuous variable in patients undergoing ventral hernia repair and showed a significant increase in the rate of in-hospital morbidity with decreasing muscle mass [20]. These findings support the view that new cut-off values are needed when defining sarcopenia in non-malignant patients. Further large-scale studies are needed to determine and clarify index values in patients with benign disease and to identify the true burden of disease [20]. A more precise value set could better identify sarcopenia in surgical patients, resulting in improved pre-habilitation and postoperative outcomes in a larger portion of patients.

The four studies included in this review revealed heterogeneity in terms of the impact sarcopenia had on postoperative complications, length of stay, return to theatre, surgical site infection and hernia recurrence [20, 27–29], although a formal meta-analysis was not possible. However, only Barnes et al. and Rinaldi et al. identified a significant increase in postoperative complications and prolonged length of stay, respectively [27, 28]. These findings, or lack thereof, are surprising considering that sarcopenia is an independent risk factor for other major abdominal surgeries.

Following this review, we believe that the heterogeneous and sparse number of significant findings can be attributed to four main sets of issues, as were demonstrated by our studies. First, the landmark axial level used to measure skeletal muscle index (SMI) varies between studies. Three of the four studies used the L3 vertebral body as their chosen landmark on axial CT, whilst Barnes et al. chose the superior border of the L4 vertebrae as their landmark [20, 27–29]. Other studies have also used the middle section of the L4 vertebral body or the L4/L5 vertebral junction as their chosen landmark.

Second, there is variability in the skeletal muscle(s) chosen for measurement on axial CT, as illustrated by the 50:50 divide between the studies in this review. Barnes et al. and Schlosser et al. measured the cross-sectional area of the psoas muscle bilaterally, whilst Rinaldi et al. and Seigal et al. measured the total skeletal muscle area of the psoas, paraspinal and abdominal musculature [20, 27–29]. Other studies have instead chosen to measure just the psoas and paraspinal muscles when calculating SMI.

Third, different techniques were used to determine skeletal muscle area between studies. Rinaldi et al. and Schlosser et al. analysed their CT scans using the Aquarius iNtuition software, which allowed for automated estimation of SMA, whilst Barnes et al. and Siegal et al. used the OsiriX software and performed semi-automated manual tracing [20, 27–29]. All four studies utilised HU in their calculations, to account for fatty tissue infiltration, bone and vasculature, however, Barnes et al. did not adjust for patient height [27].

Finally, the definition of sarcopenia varied between studies. As discussed previously, the gender-specific cutoffs used in three of the studies (< 38.5 cm^2^/m^2^ in women and < 52.4 cm^2^/m^2^ or < 54.5 cm^2^/m^2^ in males) were derived from studies assessing sarcopenia in cachectic cancer patients. Meanwhile, Barnes et al. used individually developed cutoffs, defining sarcopenia as a HUAC of less than 19.6 HU, based on the lowest quartile [20, 27–29]. Furthermore, due to HUAC being a continuous variable, Barnes et al. were able to define both the presence and severity of sarcopenia for each patient, with lower HUAC scores reflecting a more severe degree of sarcopenia [27]. These findings contribute to the growing evidence in support of the use of HUAC and other CT-assessed sarcopenic indexes, as stronger, patient-specific, predictors of postoperative complications [27, 28, 36]. HUAC measurements can be quickly derived from routine preoperative CT scans, at no additional cost and are easily performed in most clinical settings, all of which facilitate its implementation into the diagnostic workup of patients undergoing VHR or AWR [14, 27].

It is important to consider the role of sarcopenia in the context of VHR and AWR specifically. Incisional hernias remain the most common complication of laparotomies, occurring in approximately 11–30% of cases, dependent on surgical technique [37]. Known hernia risk factors include obesity, uncontrolled diabetes, active smoking, wound infection, previous hernia repairs, immunosuppression and operative technique [20, 37]. VHR and AWR are both significant physiological stressors which require a large metabolic reserve following surgery. Postoperatively, an anabolic state is required for optimal tissue repair to occur, in contrast to the catabolic strain and inflammatory state of sarcopenia. If sarcopenia was a negative determinant for adverse outcomes following VHR/AWR, rigorous preoperative management could attempt to mitigate its effect, suiting the elective nature of AWRs, which allows time for adequate prehabilitation and pre-optimisation to occur [31].

Personalised prehabilitation plans can be particularly effective in patients undergoing major abdominal surgeries [29, 31, 38, 39]. Such plans aim to optimise functional recovery and minimise postoperative morbidity [40, 41] by managing known modifiable risk factors, most commonly obesity, malnutrition, diabetes, smoking and surgical-site contamination [31, 42]. Sarcopenia is also considered to be a correctable risk factor, due to skeletal muscle mass being modifiable. A recent systematic review found exercise interventions to have a positive impact on muscle mass, function and physical performance [43], whilst a study involving patients with chronic liver disease, demonstrated a reduction in the severity of sarcopenia, following 12 weeks of combined dietary modification, nutrient supplementation and exercise [27]. Further, large-scale studies are required to elucidate the most effective treatments for sarcopenia, and over what duration, to optimise preoperative management.

To our knowledge, this is the first systematic review assessing the role of sarcopenia as a prognostic indicator in patients undergoing VHR/AWRs. Limitations of this review include the small number of articles that met the inclusion criteria and the small patient populations, which may reduce the power of our findings. Furthermore, the retrospective nature of three of the studies limits our ability to assess causation and may introduce selection bias in patient cohorts. Finally, half of the studies occurred in tertiary referral centres, reducing the generalisability of the results to other patient populations. Despite these limitations, each study in this review demonstrated several associations between sarcopenia and certain postoperative complications, which admittedly did differ, but were all trending towards significance. Finally, considering the underreported body of literature on the effect of sarcopenia on ventral hernia repairs and abdominal wall reconstructions, this review summarises the available evidence and identifies gaps in knowledge, which may guide future research.

## Conclusion

This systematic review has identified important factors contributing to the heterogeneity in results regarding the impact of sarcopenia on VHR and AWR, as found in the literature. This study emphasises the need for further, large-cohort studies, to allow for clarification of its impact on surgical outcomes and to help define different index cut-off values applicable to a benign cohort of patients, such as those awaiting VHR/AWR. Finally, further research into the reversibility of sarcopenia is required, to allow for rigorous, evidence-based preoperative management to occur and ultimately, improve surgical outcomes.

**Table 1:** Participant characteristics

References	Population size	Males, # (%)	Mean age	History of tobacco use (%)	Mean BMI (kg/m^2^)	Mean number of comorbidities	Mean SMI (cm^2^/m^2^)	Sarcopenic patients # (%)
Barnes et al. [27]	58	30 (51.7)	59.0^a^	56.9	29.7			21 (36.2)
Rinaldi et al. [28]	82	36 (43.9)	54.6		34.7	2.5	50.5^b^	21 (25.6)
Schlosser et al. [29]	1178	497 (42.2)	58.5	15.0	33.5		65.6^b^	145 (12.3)
Siegal et al. [20]	135	59 (43.7)	58.3	57.8	35.6		51.3^b^	37 (27.4)
Total	1453	622 (42.8)	57.6		33.4			224

*BMI * body mass index, *SMI * skeletal muscle index

^a^Median

^b^Measurements for core muscle size were standardized for patient height

**Table 2:** Study characteristics

References	Study period	Surgery performed	Inclusion criteria	Timing of CT scan	Core muscle analysis	Definition of sarcopenia	Definition of complications	Outcomes measured
Barnes et al. [27]	January 1st 2009—December 31st 2013	Ventral hernia repair ± other concomitant procedures	Component separation, ** ≥ **18 years old, adequate CT scans within 3 months of surgery	Preoperative	TPA (L4)	HUAC of 19.6 HU	Predefined list	Intraoperative blood loss, ICU stay, Duration, length of hospital stay, hematoma, cellulitis, seroma, delayed wound healing infection, adhesive bowel obstruction, hernia recurrence, renal failure, pulmonary failure, sepsis, number of readmissions, number of repeat surgical interventions
Rinaldi et al. [28]	July 2011—March 2013	Primary or recurrent complex ventral hernia repair	** ≥ **18 years old, adequate CT scan encompassing all abdominal wall defects and a body habitus that was sufficiently visualised within scanning field	Preoperative	TAMA (L3)	Gender-specific cutoffs (SMI < 38.5 cm^2^/m^2^ female, < 52.4 cm^2^/m^2^ male)	Predefined list	Length of stay, duration of ileus, postoperative complications, hernia recurrence, 90-day postoperative surgical site occurrences (includes seroma, surgical site infection, nonhealing and/or breakdown of incision site)
Schlosser et al. [29]	2007—2018	Open ventral hernia repair	Adequate CT scans within 1 year of surgery	Preoperative	TPA (L3)	Gender-specific cutoffs (SMI < 38.5 cm^2^/m^2^ female, < 54.5 cm^2^/m^2^ male)	Clavien-Dindo grade 3 or more (CD** ≥ **3)	Wound complications, hernia recurrence, major complications of Clavien-Dindo grade 3 or greater, reoperation, length of stay, operative time, and readmission
Siegal et al. [20]	January 2014—May 2016	Ventral hernia repair ± other concomitant procedures	VHR with or without mesh reinforcement, component separation/AWR, ≥ 18 years old, adequate CT scans within 1 year of surgery, hernia defect ≥ 2 cm	Preoperative	TAMA (L3)	Gender-specific cutoffs (SMI < 38.5 cm^2^/m^2^ female, < 52.4 cm^2^/m^2^ male)	Predefined list	Surgical site infection (SSI), surgical site occurrence (includes seroma, hematoma, dehiscence and fistula), hernia recurrence, length of stay, and in-patient morbidity (including pneumonia, respiratory/ cardiac/renal/gastrointestinal complication or failure, deep venous thrombosis, non-SSI infection, bleeding/thrombotic complication and intensive care unit transfer)

*CRLM * colorectal metastases, *TPA * total psoas area, *TAMA*  total abdominal muscle area, *LOS * length of stay, *SSO*  surgical site recurrence, *SSI * surgical site infection

**Table 3:** Hernia characteristics

References	Median hernia size	Mean hernia volume (cm^3^)	Average abdominal defect area (cm^2^)	Number of patients with previous hernia repairs, # (%)	Average number of prior hernia repairs per patient, # (%)	Primary fascial closure without mesh, # (%)	Primary fascial closure with mesh, # (%)	Concomitant procedure performed, # (%)
Barnes et al. [27]	150 cm^2^					15 (25.9)	33 (56.9)	43 (74.1)
Rinaldi et al. [28]		620.8^a^	155.8		1.4 (66%)		47 (57)	
Schlosser et al. [29]		579.9	150.8	778 (66%)	2.3			
Siegal et al. [20]	mVHWG Grade 2			85 (63%)	2.8	24 (17.8)	111 (82.2)	26 (19.3)

^a^Summed

**Table 4:** Study outcomes related to sarcopenia

References	Risk of complications	Return to theatre	Prolonged LOS	SSI	SSO	Hernia recurrence
Barnes et al. [27]	↑ (renal failure)	⟶	⟶	⟶	⟶	↑
Rinaldi et al. [28]	↑ (duration of ileus)	⟶	↑	⟶	⟶	⟶
Schlosser et al. [29]	⟶	⟶	⟶	⟶	⟶	⟶
Siegal et al. [20]	⟶	⟶	⟶	⟶	⟶	⟶

*LOS*  length of stay, *SSO * surgical site recurrence, *SSI*  surgical site infection

↑ = significantly increased risk, ↓ = significantly decreased risk, ⟶ = non-significant
